# Increased Intake of Foods with High Nutrient Density Can Help to Break the Intergenerational Cycle of Malnutrition and Obesity

**DOI:** 10.3390/nu7075266

**Published:** 2015-07-21

**Authors:** Barbara Troesch, Hans K. Biesalski, Rolf Bos, Erik Buskens, Philip C. Calder, Wim H. M. Saris, Jörg Spieldenner, Henkjan J. Verkade, Peter Weber, Manfred Eggersdorfer

**Affiliations:** 1 DSM Nutritional Products Ltd., Wurmisweg 576, Kaiseraugst 4303, Switzerland; E-Mails: peter.weber@dsm.com (P.W.); manfred.eggersdorfer@dsm.com (M.E.); 2 Institut für Biologische Chemie und Ernährungswissenschaft, Universität Hohenheim, Stuttgart 70599, Germany; E-Mail: Hans-K.Biesalski@uni-hohenheim.de; 3 FrieslandCampina, Bronland 20, Wageningen 6708 WH, The Netherlands; E-Mail: rolf.bos@frieslandcampina.com; 4 Department of Pediatrics, Beatrix Children’s Hospital/University Medical Center Groningen, University of Groningen, P.O. Box 30001, Groningen 9700 RB, The Netherlands; E-Mails: e.buskens@umcg.nl (E.B.); h.j.verkade@umcg.nl (H.J.V.); 5 Human Development and Health Academic Unit, Faculty of Medicine, University of Southampton, Southampton SO16 6YD, UK; E-Mail: pcc@soton.ac.uk; 6 Department of Human Biology, NUTRIM School for Nutrition and Translational Research in Metabolism, Maastricht University Medical Centre, Maastricht 6200MD, The Netherlands; E-Mail: w.saris@maastrichtuniversity.nl; 7 Nestlé Research Center, Vers-chez-les Blanc, Lausanne 26 1000, Switzerland; E-Mail: jorg.spieldenner@rdls.nestle.com

**Keywords:** nutrient density, vitamin, PUFA, life cycle, non-communicable diseases, obesity

## Abstract

A workshop held at the University Medical Center in Groningen, The Netherlands, aimed at discussing the nutritional situation of the population in general and the role diet plays during critical windows in the life course, during which the body is programmed for the development of non-communicable diseases (NCDs). NCDs are increasingly prevalent as our society ages, and nutrition is well known to play an important role in determining the risk and the time of onset of many common NCDs. Even in affluent countries, people have difficulties to achieve adequate intakes for a range of nutrients: Economic constraints as well as modern lifestyles lead people to consume diets with a positive energy balance, but low in micronutrients, resulting in increasing prevalence of obesity and suboptimal nutritional status. Information about nutrient density, which refers to the content of micronutrients relative to energy in food or diets, can help identify foods that have a low calorie to nutrient ratio. It thus allows the consumption of diets that cover nutritional needs without increasing the risk of becoming obese. Given the impact a nutrient dense, low energy diet can have on health, researchers, food industry and governments jointly should develop options for affordable, appealing nutrient-rich food products, which, in combination with physical activity, allow for optimal health throughout the life-course.

## 1. Introduction

In almost every country worldwide, lifespan is increasing and, in many countries, has been doing so for nearly two centuries [1]. Until the 1950s, these increases were achieved mainly through reduced childhood mortality but since then, even though mortality in those under five years of age continues to decline, gains later in life are the most important factor leading to increased longevity [2,3]. Based on current estimates, in 2040 more than one in four Europeans will be ≥65 years old and one in seven ≥75 years of age [4]. Unfortunately, for many people this gain in life years may not be a gain in years of healthy life: non-communicable diseases (NCDs) like osteoporosis, diabetes, cardiovascular diseases (CVD) and cancer are on the rise [3]. Prolonged illness frequently dominates the last decade of life [5], which has a detrimental effect on the quality of life [6]. Additionally, NCDs place a heavy burden on the economy due to their chronic nature and they affect increasingly low and middle income countries [7]. Still, an important opportunity lies in the estimate that 80% of premature deaths due to heart disease, stroke and diabetes could be prevented via modifiable factors such as improved nutrition and physical activity [8].

A workshop held at the University Medical Center in Groningen, The Netherlands, brought together experts from academia and industry to discuss the nutritional situation of the population in general and the role diet plays particularly during critical windows in the life course. Special attention was paid to the situation in infants and young children, pregnant women, the elderly, and the obese, as these are regarded as population sub-groups at a higher risk for inadequate nutrition. Dietary and lifestyle factors were identified that lead to the development of malnutrition and the concept of nutrient density—The ratio of essential nutrients to energy—Was evaluated as a tool to improve nutrition for the general population.

## 2. Current Nutritional Situation

Even though, at least in developed countries, a vast range and large quantities of foods are available independent of season or geographic location, inadequate micronutrient intakes appear to be widespread [9]. It is evident that the majority of the population does not consume what is generally regarded as a healthy diet [10]. A recent analysis of publicly available dietary surveys showed that even in affluent countries, more than three quarters of the adult population do not achieve the recommended intakes for an appreciable number of vitamins [9]. Furthermore, a significant proportion of German infants and young children aged six months to five years was found to have intakes of many vitamins below recommendations. For vitamin D, E, C and folate, this was the case for more than half of them [11]. An assessment of dietary habits in Swiss schools showed that 10% to nearly 70% of children aged 9 to 19 years old did not meet the recommendations for vitamin A, thiamine, riboflavin, vitamin B6, vitamin B12, folate, vitamin C, vitamin E, niacin and pantothenic acid [12]. Undernutrition among the elderly is a significant and neglected public health problem thought to affect approximately 10% of people over the age of 65 years old for example in the UK [13]. However, such figures only include protein-energy undernutrition and rates can be expected to be much higher if inadequate micronutrient status is also taken into consideration: A study in the US showed that intakes below the *Estimated Average Requirements* were widespread for a range of micronutrients in the elderly [14]. The situation becomes even more critical when looking at institutionalized elderly who were found to meet hardly any of the recommendations for vitamin intakes in a survey in Germany [15]. 

At the same time, obesity rates have doubled globally since 1980, resulting in 600 million obese adults in 2008 and more than 40 million overweight or obese children under the age of five years in 2011 [16]. Besides being a risk factor for developing NCDs in itself, obesity is thought to be linked to inadequate micronutrient status [17]. A recent study found higher prevalence of inadequate intake for various micronutrients in the obese compared to normal weight persons, even though some of these results might be at least partially due to underreporting of food consumption [18]. However, not only low intakes, but also impaired bioavailability and utilization of micronutrients may be involved in the inadequate micronutrient status in obesity. For some nutrients, such as iron, the low level inflammation that accompanies obesity likely decreases its absorption [19]. The lower levels of serum 25-hydroxy vitamin D found in overweight and obese subjects compared to their normal weight counterparts [20] might at least partially be due to increased sequestration of the fat-soluble vitamin in adipose tissue [21]. In addition, dieting was shown to aggravate the situation, even if the regime provides micronutrients at the levels recommended for a specific age and gender group [17]. Due to the multifactorial nature of the development of NCDs and their long latency period, it is difficult to establish a clear cause—Effect relationship between nutrition and chronic diseases [22]. Still, mounting evidence makes it increasingly clear that good nutrition throughout the whole life course is crucial in order to promote health and well-being and is even more so during specifically sensitive periods such as pregnancy, infancy, early childhood and old age.

## 3. Critical Windows throughout the Life Course

### 3.1. Programming in Early Life

Babies who are malnourished in the womb have a higher risk of dying in infancy [23], but they are also more likely to face lifelong cognitive and physical deficits and have an increased risk for developing NCDs [24,25]. The importance of nutrition early in life for health later on was recognized at the United Nations’ General Assembly in 2011: “*…[We] note also with concern that maternal and child health is inextricably linked with NCDs and their risk factors, specifically as prenatal malnutrition and low birth weight create a predisposition to obesity, high blood pressure, heart disease and diabetes later in life; and that pregnancy conditions, such as maternal obesity and gestational diabetes, are associated with similar risks in both the mother and her offspring*” [26]. Inadequate nutrition can have a profound impact on a child’s ability to rise out of poverty [27], which is particularly important as low socio-economic status is in itself a risk factor for malnutrition, obesity and consequently the development of NCDs [28].

According to the Barker hypothesis developed in the 1980s, chances of short-term survival are increased during malnourishment in pregnancy by favoring the development of vital fetal organs such as the brain at the expense of others and at the expense of changing metabolic set-points, thereby facilitating the development of metabolic impairments in later life leading to diseases [29]. Barker and colleagues proposed that inadequate nutrition early in life can alter gene expression, cell numbers and balance between cell types, organ structure as well as pattern of hormonal release and hormonal responses [30]. Gluckman *et al.* [31] suggested that the fetus makes adaptations to cope with life based on the conditions encountered during intrauterine life and that these provide an advantage for survival if similar conditions persist during adulthood, *i.e.*, scarcity of food. However, a mismatch between conditions early and later in life can lead to increased disease risk (“*Match-mismatch-theory*”). Based on data from a cohort born around the time of the Dutch famine of 1944 to 1945, it was shown that the timing of the nutritional insult is also very important in determining the health outcome: while risk of obesity and CVD was increased in persons who were nutritionally deprived early in gestation, deprivation later in gestation was associated with decreased glucose tolerance and an increased risk for hypertension [32]. The increasing evidence from epidemiologic and animal studies for this link between early nutrition and later diseases, the criticality of timing, as well as potential mechanisms involved have been reviewed elsewhere [33].

The available data indicate that the genome and the epigenome are both responsible for the adult phenotype and that the latter is greatly influenced by environmental factors such as nutrition [31]. A study in adopted persons showed a strong correlation between body weight in the offspring and in their biologic parents, particularly between the mothers and daughters, while the body weight of the adoptive parents had no significant influence [34]. This is indicative of a role for genetic factors, but also highlights the importance of the early, intrauterine environment. Similar conclusions can be drawn from an overfeeding study in twins that found a close correlation in weight gain within, and a relatively wide variation (up to a threefold difference in energy efficiency) between, the different pairs of twins [35]. Even though the mechanisms through which early diet affects the epigenome are not well understood, processes such as DNA methylation in the offspring as a result of maternal or even paternal diet have been shown in a range of studies in animals and humans [36]. A recent study in Gambia for example showed different DNA methylation patterns in blood of infants whose mothers had received periconceptional micronutrient supplements compared to those whose mothers received a placebo [37].

The development of obesity and adult disease is not only linked to under- but also to over-nutrition during early life [38], highlighting the importance of balanced nutrition. There is mounting evidence that maternal obesity alone or combined with gestational diabetes increases the risk for symptoms of the metabolic syndrome such as glucose intolerance or hypertension already during childhood [39]. This link between gestational obesity and diabetes has been studied in animal models and has been reviewed elsewhere [33]. Pre-pregnancy obesity as well as gestational diabetes seem to shift body composition towards an increased percentage of fat compared to fat-free mass in the offspring [40]. This increases their risk and, at least in the case of females, their offspring’s to become obese too. In order to revert the trend towards increasing prevalence of obesity, it is consequently important to break this vicious circle. Besides the slightly increased requirements for energy, the needs for specific macro- and micronutrients are also elevated to varying degrees during the different phases of pregnancy [41]. Therefore, a diet rich in essential nutrients at a moderate energy density to avoid both obesity and micronutrient deficiencies is crucial for the long-term health of the mother and the child.

Additionally, it has been shown that pre-pregnancy obesity and excessive gestational weight gain both decrease the success of initiation of breast feeding as well as its duration [42]. This might further increase the risk for NCDs later in life as evidence continues to emerge for beneficial effects of breast feeding on long-term health outcomes such as blood pressure and cholesterol levels as well as decreased risk of obesity and type 2 diabetes later in life [43]. Moreover, it had been proposed that breast feeding reduces the risk of obesity later in life [44], but it cannot be completely excluded (yet) that these effects are due to confounders such as maternal lifestyle, education and socio-economic status [45]. Besides providing optimal nutrition for the infant, components of human milk also support the maturation of the intestine, pancreas, the immune system and the gut microbiota [46]. Particularly the >200 human milk oligosaccharides seem to encourage the growth of commensal bacteria in the gut [47]. While the individual gut flora varies widely and undergoes rapid development following birth, the characteristic composition of an adult flora emerges during the first year of life [48]. However, this can be significantly influenced by factors such as genetics, mode of delivery at birth, duration of breast feeding and nutrition in general [49]. While many questions remain, it is becoming increasingly clear that the human microbiota has an important effect on the homeostasis of human metabolism and could play an important role in the development of obesity and NCDs such as type 2 diabetes [50] and atherosclerosis [51]. Moreover, it has been shown that there are differences in the composition of bacteria found in breast milk of obese compared to normal weight mothers [52]. 

Breast milk contains significantly higher levels of cholesterol compared to cow’s milk and it was shown that adults who had been breastfed as infants had lower total blood cholesterol and a healthier low to high-density lipoprotein ratio [53,54]. A relationship between infant feeding, adult cholesterol levels and death from ischemic heart disease had been established in a male cohort in England already 20 years ago [55]. However, despite feeding formula fortified with cholesterol, *de novo* synthesis of cholesterol remained higher than in breast fed group, albeit it was lower than in infants fed regular formula [56]. This indicates that factors other than cholesterol content are responsible for the beneficial effect of breast milk on cholesterol metabolism [56]. Moreover, the differences in cholesterol synthesis were found to disappear after 18 months [57].

Until the composition of human milk is understood in much more detail and infant formula is adapted accordingly, breast milk will continue to be the optimal diet during the first months of life [58]. As a consequence, maternal nutrition continues to be essential for the infant’s development as particularly vitamins A and D, the B vitamins, iodine and selenium, but also human milk oligosaccharides are affected by maternal diet [59,60,61]. Breast milk levels and consequently the infant’s polyunsaturated fatty acid (PUFA) status also depend strongly on maternal intake and, given the high rate of brain growth in infants, increased intakes of particularly long chain PUFAs might have beneficial effects [62]. Stunting in early childhood, an indicator for suboptimal nutrition, not only increased the risk for low educational attainment and poverty in adults, but was also linked to birth of first child at younger age and higher number of pregnancies [63], which increases the risk of inadequate nutrition during pregnancy even in developed countries: Nearly 30% of pregnant German women with short birth spacing or expecting twins had serum retinol levels below what is regarded as adequate, even though they had a high to moderate socioeconomic background [64]. Similarly, a study in Dutch women found that the levels of docosahexaenoic acid decreased with every new pregnancy [65]. 

Consequently, ensuring a good quality diet throughout pregnancy as well as during lactation is crucial to break the intergenerational cycle of obesity and inadequate nutrition. There is increasing evidence that postnatal nutrition also plays a role in determining the risk for the development of NCDs later in life: various studies showed that individuals who were small at birth and had the fastest postnatal growth had highest risks for NCDs later in life [66,67,68,69,70,71,72]. High-energy diets with limited nutrient density seem to favor the synthesis of adipose tissue, resulting in increased accumulation of fat rather than muscle tissue and bone mass [73]. Obesity in childhood not only increases the risk of remaining obese throughout adulthood, but obese children frequently already present with other risk factors for CVD such as high blood pressure or cholesterol and type 2 diabetes [3].

### 3.2. Adulthood and Old Age

Bruce Ames formulated the so-called triage theory, which postulates that during times of nutritional scarcity, evolutionary priority was given to short-term survival at the expense of damage evident in older age [74]. Studies increasingly indicate that some of the adverse effects can be countered by adequate nutrition: A recent RCT with a follow-up of more than a decade showed that long-term supplementation with multi-micronutrients significantly reduced overall cancer risk [75]. According to the authors, similar effects in the same subjects on the risk for cardiovascular events, eye diseases and cognitive decline will be published separately. Low vitamin D status was found to increase the risk of CVD events, particularly in hypertensive persons [76]. Similarly, another study found that low vitamin D levels were linked to increased all-cause and CVD mortality [77].

Inadequate levels of a range of vitamins are linked to cognitive decline in the elderly: Low serum vitamin D levels were linked to increased risk for the development of various forms of dementia [78,79,80,81]. Even though some inconsistencies exist, a link between low levels of various B vitamins and cognitive decline has been proposed [82] and it was subsequently shown that a supplement containing folate, vitamin B6 and B12 could slow down the progression of brain atrophy in elderly with mild cognitive impairment [83]. In addition, serum levels of the antioxidant vitamins A, C and E were shown to be negatively associated with the development of mild cognitive impairment and Alzheimer’s disease [84]. Moreover, there is some evidence that improving micronutrient status has a beneficial effect on mood in elderly subjects [85].

The relevance of vitamin inadequacies for life quality and health care cost has been studied in most detail for vitamin D: among patients in a medical intensive care unit, vitamin D deficiency at admission was as high as 78% and 25-hydroxy vitamin D levels were found to correlate inversely with mortality after ≥2 days in the hospital [86]. Vitamin D status was inversely related to hospital stay and 25-hydroxy vitamin D levels were found to decrease further during the stay at the surgical intensive care unit [87]. As a consequence of the increased time spent at the hospital, treatment cost more than doubled for people with severe vitamin D deficiency [87]. 

Old age and the accompanying pathologies result in a multitude of physiological, psychological and social changes. Older people tend to eat alone more often than younger ones, which was shown to result in decreased food intakes [88]. Due to physical disabilities, dementia, depression or other psychological factors, they are less capable of preparing meals for themselves [89]. A recent study showed that disability and chronic illness significantly increased the odds of a diet characterized by low variability [90]. Moreover, decreased effectiveness to detect and react to hunger further increases the risk of malnutrition [88]. Appetite tends to be lower due to declining taste and smell sensitivities, various pathological conditions or medications, and impaired chewing due to tooth loss and ill-fitting dentures [91]. Reduced secretion of saliva or the intake of certain drugs makes swallowing more difficult [92]. All these factors increased the risk of an inadequate nutritional intake.

Changes in body composition as part of the aging process are associated with an increased risk for malnutrition as they lead to decreased energy needs [93] whereas the requirements for micronutrients remain similar to those of adults in general [94,95,96] or, in the case of vitamin D, increase [97]. A study in healthy elderly found intakes below the *Estimated Average Requirements* for most participants for vitamin D and calcium and for around half the participants for vitamin B6, folate, magnesium and zinc, while energy intake corresponded to the recommendations [98]. This highlights the importance of nutrient dense foods for population groups with low levels of activity and therefore reduced energy intakes [99].

## 4. Improving Nutrition along the Life Course

An effective way of covering the needs for essential nutrients while avoiding obesity seems to be a varied diet, as diversity was found to correlate positively with the quality of the diet, but also with anthropometric indicators of physical development in children [100]. Various efforts have been made to convince consumers of the importance of a balanced diet and following dietary guidelines was shown to be a good strategy for obesity prevention, as good adherence lead to lower six-year-weight gain in French adults [101]. However, there is clear evidence that consumers do not adhere to dietary recommendations as they tend to over-consume foods full of “empty calories”, while not eating enough fruits, vegetables, whole grains and milk [10]. A survey by the US Departments of Agriculture and Health and Human Services showed that people consistently tended to eat too much of food categories with recommended maximal intake (e.g., solid fats and added sugars) and too little of foods where minimal amounts were advised (e.g., fruits and vegetables) [102]. This seems to be true for most parts of the world as a recent study reported low intakes of fruits and vegetables for 58% to 88% adults across all geographic clusters [103]. This is particularly critical as energy dense diets appear to play an important part in the obesity epidemic: A series of experiments in humans showed that energy density rather than fat content determined overeating [104]. However, in practice energy dense foods are closely linked to high fat foods [105]. Prentice and Jebb reason that humans have a weak innate ability to adapt food intake to its energy density as this had not been important with the diets in early agricultural societies [104]. Several *ad libitum* randomized controlled trials have shown that with a 10% reduction in fat content in the diet, on average 3 kg of body weight lost is observed [106].

The globally increasing consumption of sugar-sweetened beverages has been proposed as a factor that contributes to the raised obesity rates [107]. The rationale is that liquid energy, for example from soft drinks, is much less satiating than an equal amount of energy in solid foods, which increases the risk of a positive energy balance [108]—Even though the energy density is not necessarily particularly high due to the high water content. A recent review concluded that there was convincing evidence that sugar-sweetened beverages exacerbated the risk of becoming obese in individuals with a genetic predisposition towards increased body weight [109]. However, another one found inconsistent results for children, adolescents as well as adults once the data were adjusted for energy intake and physical activity [110]. The association between intake of such beverages and the risk of CVD also seems to be at least partially due to their high contribution to total energy intake while providing very limited amounts of essential nutrients [111]. However, their impact on blood glucose and consequently insulin levels might also play an important role [111]. A recent study found an association of consumption of added sugar with diastolic blood pressure as well as with serum triglycerides in a cohort of US children [112]. Similar results were found in studies looking for an association between consumption of sugar-sweetened beverages and metabolic syndrome [113,114,115]. In addition, an association has been proposed between sugar-sweetened beverages and the development of type 2 diabetes, showing a 30% increased risk for the participants with the highest compared to the lowest intakes [116]. A recently published study found that higher intakes of sweetened soft drinks were linked to insulin resistance and increased leptin levels [117]. Moreover, increased consumption of sweetened soft drinks was linked to a decreased intake of calcium as well as other nutrients [118], which is in itself a risk factor for adverse health outcomes. Similar conclusions were reached in a study that found higher risks of inadequate intakes of various vitamins and minerals in individuals with higher intakes of added sugars [14]. Given their importance as contributors to obesity and related health problems despite relatively low energy density due to the high water content, it has been proposed that specific calculation schemes for energy density of beverages are needed [119].

Moreover, while the consumption of energy dense, nutrient poor foods appears to contribute to increased obesity prevalence, this development is also facilitated by a range of lifestyle and environmental factors: low access to supermarkets and the abundant availability of take-out meals with high sugar sweetened beverages were found to contribute to an obesogenic environment [120]; sleep deprivation, often a result of a hectic lifestyle, appears to affect appetite regulation and thereby contributes to weight gain [121]; and increased energy intake was also caused by cognitively demanding, typically computerized, work combined with the pressure to excel which has become an integral part of modern society [122]. Some of these lifestyles are unlikely to disappear anytime soon as they are thought to increase competitiveness [122]. Therefore, solutions are needed that accommodate both modern life styles and optimal health and at least part of this is likely to be via diets with increased nutrient density. 

### Improving Dietary Quality through Micronutrient Density

Various indicators to measure nutrient density have been proposed and these were reviewed in detail elsewhere [123]. The nutrient density approach is promising as it has been shown that consuming nutrient dense foods was associated with a modestly decreased risk of CVD, diabetes and all-cause mortality [124]. One example for an indicator of nutrient density is the Nutrient Rich Food Index (NRF), which can be used to rank foods, single meals or diets according to their nutritional value and can help consumers improve their diet [125]. When applied to the diets of an elderly Dutch cohort, there was an inverse correlation with all-cause mortality while no association with CVD incidence was found [126]. This latter finding might reflect the importance of past rather than present diet, as more attention might be paid to the quality of the diet once a disease is diagnosed: The authors report that participants with hypertension and diabetes actually had a higher NRF score than their peers [126].

Economic constraints were identified frequently as a main driver towards a lower quality diet as foods rich in micronutrients (*i.e.*, nutrient dense foods) are frequently more expensive than energy dense options [123,127]. It has been proposed that the risk of becoming obese is not so much linked to the consumption of individual foods or nutrients, but rather to the cost of the complete diet [128]. Therefore, the cost variable may help explain partially why lower income groups fail even more to comply with dietary guidelines and why they have the highest likelihood to consume diets associated with adverse health outcomes [129]. Income has been identified as a major factor that determined the quantity as well as the variety of fruits and vegetables consumed [130,131]. Nutrient density had a positive correlation with household budget available for food as the intake of fruits, vegetables, meat, fish and eggs decreased, while cereals, added fats and sweets increased with decreasing household budget [132]. Higher education and income were positively and significantly associated with the nutrient density measure, but these effects were greatly attenuated with the inclusion of the cost variable in the model [133].

Given these observations, it is not surprising that the recent economic crisis had a critical impact on the prevalence of malnutrition [134]. Experience from the East Asian economic crisis a decade earlier showed that health and nutrition were affected via a decrease in household purchasing power due to rising food prices, widespread unemployment and increased cost for health care [135]. Even in more affluent countries, the financial crisis lead to reduced household incomes, increased out of pocket spending for health care due to austerity measures and consequently reduced means available to spend on a healthy diet [136]. In the United States, a marked increase in the number of households categorized as food insecure or even very insecure was reported in 2008 and since then the figures have remained at around 18 million and 7 million, respectively [137]. Since in general the diversity and consequently the nutrient density of the diet is reduced before energy intake is decreased, these developments can be expected to increase the risk of micronutrient deficiencies [138]. Furthermore, food insecurity is thought to contribute to the development of obesity, even though the causal link is not entirely clear [139,140].

Given the importance of available income for dietary choices, nutrient density indices can also help to identify foods with good nutrient to cost ratio [141,142]. A study comparing nutrient density with cost confirmed that the former tends to be associated with a higher price per 100 g [143]. However, they also found that this was not the case for all fruits and vegetables and identified around 30 which had a better nutrient to cost ratio, including for example fresh oranges or carrots, but also canned or frozen vegetables such as tomatoes or green peas [143]. Milk and milk products are good sources of calcium, but also protein and they, as well as beans and eggs, are other high quality foods available at reasonable prices [144]. Moreover, it has recently been shown that there is no correlation between cost and the quality of the diet in children and adolescents whose diets were classified as low quality [145]. In other words, there is a potential to decrease energy and increase nutrient density of these diets without affecting the total cost by replacing products providing only empty calories with for example fruits or vegetables [146].

When consumer acceptance measured as frequency of use was included in the calculation, white potatoes emerged as a food that combined high nutritional quality with a relatively low energy density and high affordability and acceptance [147]. Particularly in developing countries, milk from cows or other mammals has been identified as an important source of protein as well as various micronutrients needed for growth and development in children [148]. In areas where the locally available diet makes it difficult to increase micronutrient density of the foods by increasing diversity, specifically targeted foods are a promising tool especially in the short term: A recent review concluded that fortified milk and cereal products provided an effective way of reducing the risk of anemia in young children in developing countries [149].

It has frequently been postulated that taxes should be used to guide people’s dietary choices by increasing the cost of unhealthy foods. However, such public health measures can only be effective if more nutrient and less energy dense, affordable alternatives are available. Consequently, the development of tasty foods with a high micronutrient density at a reasonable price is essential whether or not fiscal measures are implemented to improve the quality of the general population’s diet. Given the complexity and interconnectedness of choices related to foods and their prices, especially for households with a tight budget, such measures need to be well thought through. Also, it appears that our craving for a food or nutrient is not driven by a physiological need, but rather via a separate hedonistic system, which can encourage overconsumption of certain tasty foods [150]. When designing a meal or food with low energy and high nutrient density, it is consequently important to find a balance between acceptance, nutrient content and appetite control as high palatability can led to energy overconsumption even if caloric density is low [151]. A promising example for this approach is the development of affordable, nutrient dense lunch bags for schools in Canada [122]: Thanks to a good balance between palatability and feeling of satiety, they were shown to result in decreased spontaneous energy intake [122].

## 5. Conclusions

Improvements in health care and other developments in recent years have led to an increase in global life expectancy, resulting in a dramatic shift in demography [2]. However, at the same time, increasing rates of NCDs frequently affect the quality of life during at least the last decade of life [6]. Moreover, the high prevalence of obesity, increasingly at an early age, accompanied by symptoms of NCDs already in children makes a reverse in the trend of life expectancy conceivable [152]. The complex relationship between nutrition, obesity, microbiota, NCDs, but also poverty or low socio-economic status is not entirely understood. The opportunity is to recognize how nutrition modulates health and to identify, develop and implement nutritional solutions promoting a healthy life.

**Figure 1:**
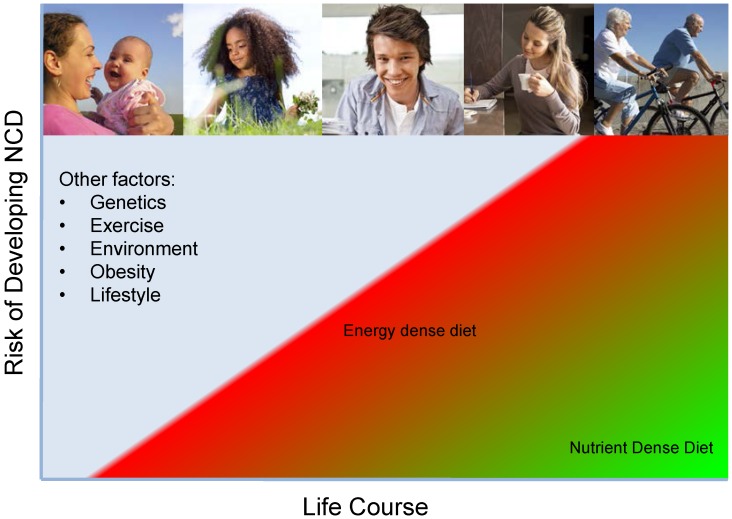
Decreasing energy and increasing nutrient density of the diet throughout the life course is thought to have a beneficial effect on the risk of developing NCDs.

Despite the wide range of factors affecting health, it seems likely that shifting diets from energy dense to nutrient dense will have an significant beneficial effect on the risk of developing NCDs along the life course and helping to keep life expectancy, but also quality of life, high (Figure 1). The nutrient density approach can be a valuable tool in nutrition education and dietary guidance. Solutions have to take into account the changing needs and windows of opportunities over the life course. While a range of stakeholders such as industry, government and academia have a responsibility in supporting the public through transparent labeling, smart regulations and the improved accessibility to nutrient rich foods, the responsibility of the individual as well as their right to freedom of choice cannot be neglected.

The goal of this workshop was to present available evidence for the benefits of healthy nutrition throughout the life course and to discuss how the concept of nutrient density can help resolve some of the problems arising from the demographic and lifestyle changes currently underway. In a next step, scientists and the food industry jointly should develop affordable, nutrient-rich products that can address nutritional deficits in consumers’ diets. One of the challenges, but also opportunities, for the food industry is to provide appealing, affordable products which enable people to enjoy diets which, in combination with physical activity, allow for optimal health throughout the life-course in line with modern lifestyles. Convincing food, beverage and condiment producers to make their products healthier by enhancing nutrient content and replacing or reducing public health sensitive nutrients such as saturated fats, trans-fatty acids, sodium or sugar would be a major step forward along the road to good nutrition. This approach could make a major contribution to tackling both over- and undernutrition and its implementation will be the topic of a follow-up workshop.
